# Definition of the transcriptional units of inherited retinal disease genes by meta-analysis of human retinal transcriptome data

**DOI:** 10.1186/s12864-023-09300-w

**Published:** 2023-04-18

**Authors:** Karla Alejandra Ruiz-Ceja, Dalila Capasso, Michele Pinelli, Eugenio Del Prete, Diego Carrella, Diego di Bernardo, Sandro Banfi

**Affiliations:** 1 Telethon Institute of Genetics and Medicine (TIGEM), Via Campi Flegrei, 34, 80078 Pozzuoli, Italy; 2 Dipartimento di Scienze e Tecnologie Ambientali, Biologiche e Farmaceutiche, Program in Molecular Life Science, University of Campania “Luigi Vanvitelli”, Via Vivaldi, 43, 81100 Caserta, Italy; 3 Scuola Superiore Meridionale (SSM, School of Advanced Studies), Genomic and Experimental Medicine Program, University of Naples “Federico II”, Largo S. Marcellino, 10, 80138 Napoli, Italy; 4 Chemical Engineering, University of Naples “Federico II”, Piazzale Tecchio, 80, 80125 Napoli, Italy; 5 Department of Precision Medicine, University of Campania “Luigi Vanvitelli”, Via de Crecchio, 7, 80138 Napoli, Italy

**Keywords:** Inherited retinal disease, RNA-seq, Transcriptome, Alternative splicing, Human retina

## Abstract

**Background:**

Inherited retinal diseases (IRD) are genetically heterogeneous disorders that cause the dysfunction or loss of photoreceptor cells and ultimately lead to blindness. To date, next-generation sequencing procedures fail to detect pathogenic sequence variants in coding regions of known IRD disease genes in about 30–40% of patients. One of the possible explanations for this missing heritability is the presence of yet unidentified transcripts of known IRD genes. Here, we aimed to define the transcript composition of IRD genes in the human retina by a meta-analysis of publicly available RNA-seq datasets using an *ad-hoc* designed pipeline.

**Results:**

We analysed 218 IRD genes and identified 5,054 transcripts, 3,367 of which were not previously reported. We assessed their putative expression levels and focused our attention on 435 transcripts predicted to account for at least 5% of the expression of the corresponding gene. We looked at the possible impact of the newly identified transcripts at the protein level and experimentally validated a subset of them.

**Conclusions:**

This study provides an unprecedented, detailed overview of the complexity of the human retinal transcriptome that can be instrumental in contributing to the resolution of some cases of missing heritability in IRD patients.

**Supplementary Information:**

The online version contains supplementary material available at 10.1186/s12864-023-09300-w.

## Background

The human retina delivers visual information and transduces it into neural signals and is composed of more than 60 different cell types spread across seven cell classes [1]. Inherited retinal diseases (IRDs) are a heterogeneous group of visual disorders characterized mainly by dysfunction or loss of photoreceptor cells, which can ultimately lead to blindness [2]. The inheritance pattern of IRDs is heterogeneous, including autosomal dominant, autosomal recessive, X-linked and mitochondrial patterns [2]. To date, 280 genes, listed in RetNet [3], have been either primarily linked to IRDs or shown to represent susceptibility factors. IRD genes encode proteins involved in diverse functions, including phototransduction and visual cycle, transcription regulation, splicing, and primary cilia organization [4]. From the clinical viewpoint, several criteria, such as the age of onset, progression rate, presence of extra-ocular symptoms, and the primary retinal cell target, contribute to the classification of IRDs [2].

In recent years, next-generation sequencing (NGS) technologies have revolutionized the diagnostic processes and contributed to an extraordinary advance in our knowledge of the pathogenic mechanisms underlying Mendelian diseases, including IRDs [5]. Currently, it is possible to identify the genetic causes of IRDs in about two-thirds of analysed cases [6] while the other one third remain unsolved because they do not harbour *bona fide* pathogenic variants in coding regions of already known IRD genes. This missing heritability may be due to the presence of a) mutations not readily detectable by genomic NGS-based procedures, b) novel IRD genes, or c) unknown functional elements belonging to known IRD genes. Concerning the last of these, transcriptome analysis by RNA-sequencing (RNA-seq) has a high potential to provide new insights into the possible sources of the still undiagnosed IRD cases [7–9].

Multiple mRNA isoforms produced by alternative splicing (AS) of single genes can account for the proteome diversity that contributes to organismal complexity [10]. AS events include exon skipping, exon elongation, novel exons, and intron retention. Around 9% of IRD-causing mutations affect splicing, as reported in the case of cone-rod dystrophy, Usher syndrome (USH), retinitis pigmentosa (RP), and Leber Congenital Amaurosis (LCA) [11–14]. Interestingly, it was suggested recently that the extent of AS-generated transcript diversity in the retina is higher than expected [12]. Therefore, identifying previously undetected AS events that can generate novel isoforms in the human retina could be instrumental in providing a better understanding of retinal function and disease mechanisms. Previous efforts to define the genomic structure of specific IRD genes identified novel alternative transcripts in *CERKL* [15], *BBS8* [16]*, RPGR* [17], and *RGR* [18], thus improving our understanding and diagnosis of retinal diseases*.* It is therefore important to extend such studies by exploiting comprehensive and integrated RNA-seq datasets derived from multiple human retina samples to systematically define the genomic organization of all IRD genes. We retrieved the two largest, publicly available RNA-seq datasets from non-visually impaired human donors to gain insight into the genomic organization and transcript composition of known IRD genes*.* Here, we describe the most comprehensive catalogue of IRD gene transcripts in the human retina and provide a detailed annotation of alternatively spliced isoforms. We also estimated for each analysed IRD gene the relative expression contribution of the corresponding transcripts and their coding potential. Finally, we performed experimental validation of a subset of newly predicted IRD transcripts to further corroborate the reliability of our bioinformatics pipeline. The generated data are publicly available on a web-based database. We believe this resource can enable the identification of novel disease-causing mutations in unsolved patients.

## Results

### General architecture of human IRD genes

A total of 177 bulk RNA-sequencing (RNA-seq) human retina data from non-visually impaired retinal post-mortem donors were retrieved from two different datasets. The first dataset (TIGEM) contains 50 human retina samples [8] whereas the second dataset (NEI) contains 127 samples [9, 19]. After quality control, we retained 161 RNA-seq samples for further analysis. Over 70% of the reads were successfully mapped to the human genome reference hg38.v98 using STAR.

**Fig. 1:**
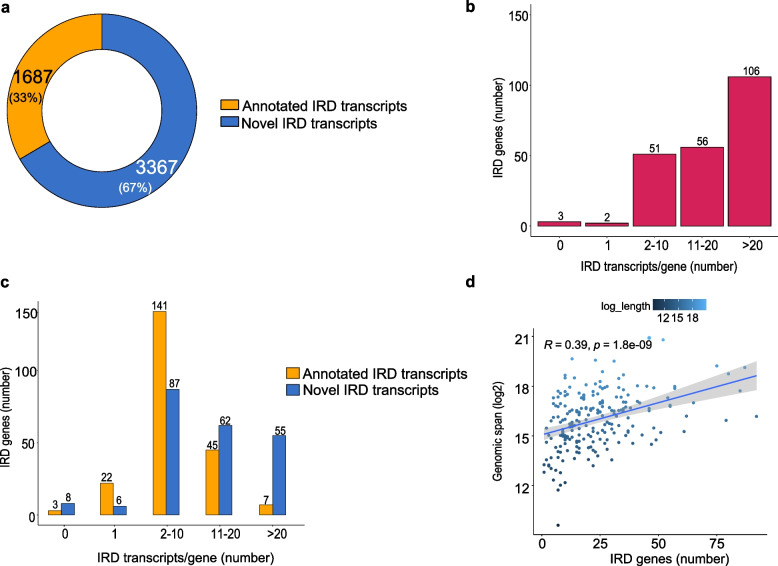
Analysis of IRD gene transcriptional units. **A** Number of already annotated and novel IRD transcripts with a scaled median TPM > 1 belonging to the analysed IRD genes. **B** Distribution of IRD genes according to their number of total distinct transcripts. **C** Distribution of IRD genes according to their number of already annotated transcripts (orange bars) and newly predicted transcripts (blue bars). **D** Correlation between the total number of distinct transcripts per gene and the genomic span of the corresponding gene. X-axis, number of IRD genes; Y-axis, genomic span (bp) in log2

We focused our analysis on 218 nuclear-encoded genes with a primary pathogenic role in monogenic forms of IRDs, selected from RetNet [3] (Supplementary Dataset File 1). Using StringTie, we identified a total of 5,650 transcripts, 596 of which were filtered out for low expression (less than one median transcript per million—TPM). Therefore, we retained a set of 5,054 transcripts for further analyses (Fig. 1a, Supplementary Dataset File 2). None of the transcripts from the *DTHD1, GDF6,* and *SPP2* genes passed the 1 TPM filter cut-off, so we discarded these genes from further analyses.

On average, we identified 23.18 transcripts per gene, of which 15.44 were newly predicted, and 7.74 were already reported (Supplementary Dataset File 3). Overall, our data show that almost 50% of IRD genes have more than 20 distinct transcripts each (Fig. 1b). The majority of IRD genes harbour between two to ten transcripts each, both already annotated and newly predicted (Fig. 1c). Moreover, we calculated the Spearman correlation between the genomic span of each gene and the corresponding number of transcripts and found a modest correlation (R = 0.39, *p* = 1.8e − 09) (Fig. 1d). Hence, we concluded that the number of distinct transcripts per gene does not substantially depend on the underlying genomic size.

**Fig. 2:**
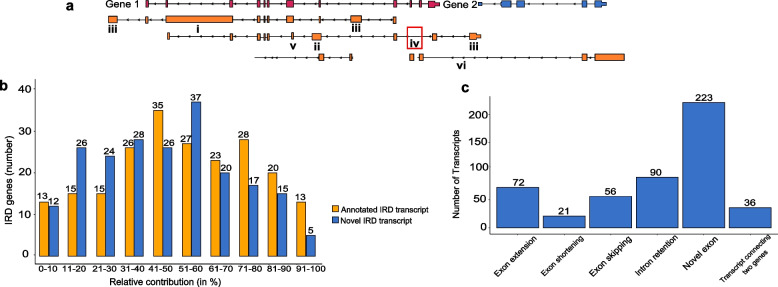
Novel transcripts description. **A** Schematic representation of different types of newly identified transcripts, which result from alternative splicing events that lead to intron retentions (i), exon extensions (ii), novel exon inclusion (iii), exon skipping (iv) and exon shortening (v). Example of a connection between two transcriptional units is represented by isoform (vi) that connects Gene 1 and 2. **B** Relative contribution to overall gene expression by annotated and novel IRD transcripts. X axis, relative contribution (in %) of gene expression in bins of 10, Y axis, number of IRD genes. **C** Structural classification of novel HEITs (highly expressed IRD transcripts). New exons are the most predominant feature, followed by intron retentions, while exon shortenings are the least common events

Transcripts were characterized as novel if they presented features that were not previously observed in the already annotated ones. These features could be one or more of a) intron retention, b) exon extension, c) novel exons, d) exon skipping, e) exon shortening, and f) connections with other transcriptional units (Fig. 2a).

### Relative contribution of IRD transcripts to corresponding gene expression

To verify the biological relevance of all the IRD transcripts, and particularly of the putative novel ones, we sought to determine their relative expression contribution (see also Methods) with respect to the corresponding transcriptional unit as a whole, i.e., the abundance of any given transcript compared to the sum of transcripts produced from the same gene. Supplementary Dataset Files 2 and 4 show the estimated expression contributions for all the IRD transcripts with over one median TPM. Figure 2b reports the distribution of analysed IRD genes based on the expression contribution of annotated and newly predicted transcripts.

We established a threshold of 5% contribution to overall gene expression of the corresponding gene and retrieved 936 IRD transcripts, defined as "highly expressed IRD transcripts" (HEITs): 501 of these were already annotated, whereas 435 were newly predicted transcripts. For only one gene, namely *INVS*, we could not identify any transcript with a relative contribution higher than 5% (Supplementary Dataset Files 5 and 6).

As expected, already-known transcripts account for the highest gene expression for most genes. For instance, annotated transcripts accounted for 100% of overall gene expression in the case of the *NYX* and *TOPORS* genes. Similarly, annotated transcripts account for more than 95% of the overall expression in the case of the *GNAT2, RHO, USH1G, NDP, PDE6H,* and *RAB28* genes.

On the other hand, the contribution of the putative novel HEITs to the overall gene expression was remarkably high in some genes. For 13 IRD genes, the sum of the overall gene expression of novel HEITs was predicted to account for greater than 70% of the corresponding gene expression (Table 1). Novel exons and intron retentions were the most common features among novel HEITs with 223 and 90 events, respectively (Fig. 2c).

**Table 1:** IRD genes in which novel HEITs cumulatively account for greater than 70% of overall expression

Gene	Contribution (%)
*NEUROD1*	95.13
*INPP5E*	90.75
*GUCY2D*	83.65
*RGS9BP*	83.09
*ARL3*	81.53
*ATF6*	80.93
*C8orf37*	80.77
*KIF3B*	77.65
*LCA5*	76.67
*BBS10*	75.50
*PRPH2*	74.29
*GRK1*	73.46
*PDE6C*	72.72

### Coding potential of novel isoforms

To assess the coding potential of the newly identified transcripts, we searched for Open Reading Frames (ORF) spanning at least 200 nucleotides. We focused on the putatively more abundant novel HEITs (*n* = 435) and identified 1,709 putative ORFs. We then compared these ORFs with the canonical sequences of the corresponding proteins. Overall, we found that the vast majority of the identified ORFs from novel HEITs are predicted to either display the same ORF as the annotated transcripts (*n* = 898) or constitute truncated products of annotated IRD proteins (n = 345). The remaining 466 identified ORFs were novel. Of these, 19 ORFs, while spanning the entire length of the annotated ORF of the corresponding gene, diverged significantly due to exon skipping or novel exon inclusion events (Table 2). We further analysed these 19 ORFs using the HMMER and CDD databases to identify possible changes in their protein domain composition compared to their canonical annotated versions. However, we did not detect any substantial differences in protein domain representations in the newly identified ORFs. Overall, protein domains were preserved in most of the novel ORFs with an increase or decrease in length caused by the novel features.

**Table 2:** List of novel ORFs predicted from novel HEITs that significantly alter the corresponding annotated ORF

Gene	Transcript ID	Refseq of annotated ORF	Novel transcript feature	Novel ORF feature
*ATF6*	MSTRG.2704.13	NM_007348.4	Connections with other transcriptional units	179 amino acids added at the N-terminus
*BBS1*	MSTRG.7009.36	NM_024649.5	Connections with other transcriptional units	127 amino acids added at the N-terminus
*CERKL*	MSTRG.23909.5	NM_201548.5	Alternative 3' coding exon	31 amino acids added at the C-terminus
*KCNV2*	MSTRG.40364.4	NM_133497.4	Alternative 3' coding exon	92 amino acids added at the C-terminus
*LRAT*	MSTRG.31449.4	NM_004744.5	Alternative 5' coding exon	20 amino acids added at the N-terminus
*PRCD*	MSTRG.18347.19	NM_001077620.3	Alternative 5' coding exon	Generation of a different protein product
*RBP3*	MSTRG.4643.1	NM_002900.3	Alternative 5' coding exon	45 amino acids added at the N-terminus
*RBP4*	MSTRG.5275.2	NM_006744.4	Alternative 3' coding exon	67 amino acids added at the C-terminus
*CNGA3*	MSTRG.22934.5	NM_001298.3	Skips canonical exon 3	38 amino acids removed
*CWC27*	MSTRG.32403.4	NM_005869.4	Novel exon predicted in intron 11	7 amino acids added
*KIAA1549*	MSTRG.38096.12	NM_001164665.2	Novel exon predicted in intron 12	16 amino acids added
*MAK*	MSTRG.34222.19	NM_001242957.3	Alternative 3' coding exon	2,163 amino acids added at the C-terminus
*PDE6C*	MSTRG.5276.12	NM_006204.4	Alternative 5' coding exon	14 amino acids added at the 5' end
*PRDM13*	MSTRG.35426.2	NM_021620.4	Skips canonical exon 2	44 amino acids removed
*RGR*	MSTRG.5132.34	NM_001012720.2	Skips canonical exons 2 and 3	52 and 45 amino acids removed
*UNC119*	MSTRG.17051.7	NM_005148.4	Skips canonical exon 2	38 amino acids removed
*ZNF408*	MSTRG.6569.5	NM_024741.3	Alternative 5' coding exon	9 amino acids added at the N-terminus
*ZNF423*	MSTRG.15493.3	NM_001379286.1	Alternative 5' coding exon	80 amino acids added at N-terminus
*ZNF423*	MSTRG.15493.5	NM_001379286.1	Novel exon predicted in intron 1	23 amino acids added

To comprehensively analyse the structural impact of these novel transcripts, we also carried out a structural model analysis by AlphaFold2 and ColabFold software. Protein modelling showed that the HEITs involving exon skipping were characterized by removing helices and unstructured loops. In contrast, the HEITs with novel exons led, in most cases, to the addition of unstructured loops (62.50%) followed by helices (18.75%) and sheets (6.25%), suggesting that these novel exons can modify protein surfaces (Supplementary Fig. S1).

### Experimental validation

**Fig. 3:**
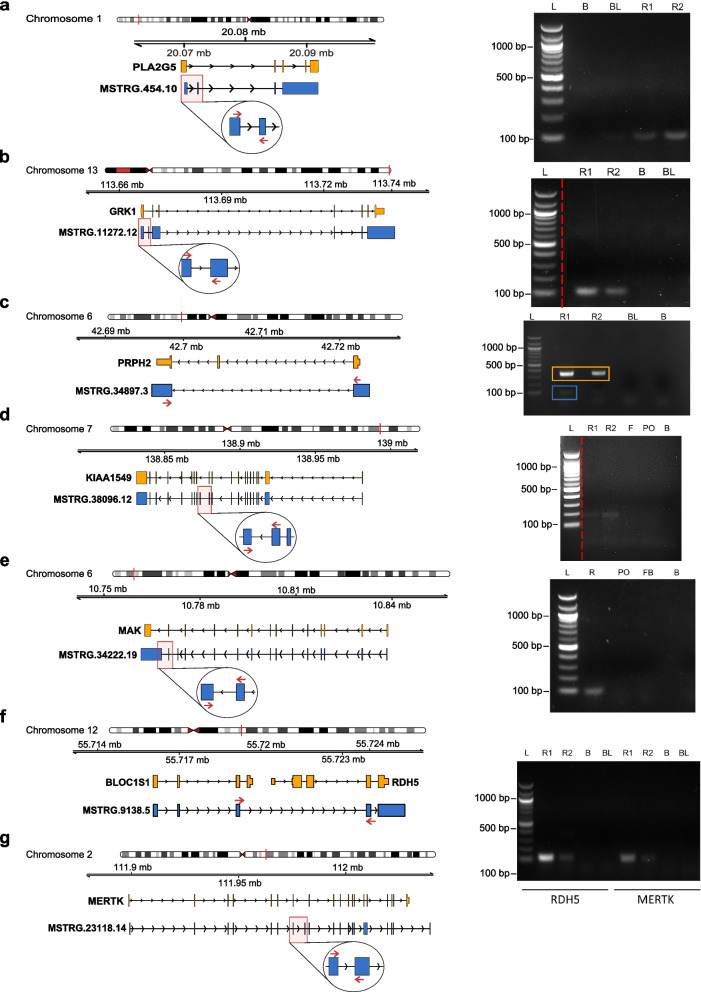
RT-PCR validation of a subset of HEITs. Left panels: schematic representations of each analysed transcript (depicted in blue) with respect to its corresponding canonical form, as defined in the Ensembl database (depicted in orange). Forward and Reverse oligonucleotide primers used in RT-PCR experiments to validate the distinguishing features of the novel transcripts are depicted as red arrows in the higher magnification insets (circles). Right panels: Agarose gel electrophoresis of RT-PCR products. **A** Validation of a *PLA2G5* transcript containing a novel exon. **B** Validation of a *GRK1* transcript containing a novel exon. **C** Validation of a *PRPH2* transcript containing a novel exon skipping event. The RT-PCR product corresponding to the canonical *PRPH2* transcript is indicated in orange, whereas the product corresponding to the novel isoform is in blue. **D** Validation of a *KIAA1549* transcript that harbours a novel exon. **E** Validation of a *MAK* transcript that contains an alternative last coding exon). **F** Validation of an *RDH5* transcript that is connected with the adjacent *BLOC1S1* transcriptional unit. **G** Validation of a *MERTK* transcript that contains a novel exon. Please note that the images showing the RT-PCR results of *GRK1* (B) and *KIAA1549* (D) were cropped and reorganized for the sake of clarity (source data are shown in Supplementary Fig. S2). L, 1000 bp ladder; B, Blank; BL, Blood; R, Retina; R1, Retina1; R2, Retina2; PO, Podocytes; F, Fibroblasts

We experimentally validated a subset (*n* = 15) of newly identified HEITs by RT-PCR on total RNA extracted from human retinas and other tissues used as controls. We selected these transcripts based on the following features: a) a minimum median TPM of 10; b) the presence of one of the following features: novel exon inclusion, exon skipping, or connections with other transcriptional units; c) significant impact on the coding potential of the corresponding gene product. Almost half of the tested HEITs (*n* = 7 out of 15) were confirmed by RT-PCR and Sanger sequencing of the generated products. In most of these cases, the novel HEITs were detected only in the retina and not in non-retinal tissues, thus supporting their retina-specific expression. However, in the case of the *PLA2G5* gene, a faint band was observed in blood, in addition to the retinal bands (Fig. 3a). Figure 3 summarises the structure of the validated HEITs and the RT-PCR products.

The *Phospholipase A2 Group V* gene (*PLA2G5*) is a member of the secretory phospholipase A2 family that catalyses the hydrolysis of membrane phospholipids. Mutations in *PLA2G5* are responsible for an autosomal recessive form of benign fleck retina (OMIM: 228,980) [20]. We predicted the presence of 24 putative novel isoforms for this gene, four of which had an expression contribution higher than 5%. We validated the putative isoform MSTRG.454.10 (median TPM of 143.28; 17% contribution to overall *PLA2G5* levels), which differed from the canonical transcript by the presence of a novel exon and an intron retention (Fig. 3a).

Similarly, the *G Protein-Coupled Receptor Kinase 1* gene (*GRK1)* was predicted to have 13 novel isoforms, two of them contributing to more than 5% of overall gene expression. We validated by RT-PCR the transcript MSTRG.11272.12 (Fig. 3b), which has a novel exon and an intron retention, with a median TPM of 476.20 and a contribution higher than 15% to overall *GRK1* expression. *GRK1* encodes a G-protein-coupled receptor kinase subfamily. Variants in this gene are associated with recessive congenital stationary night blindness Oguchi type-2 (OMIM: 258,100) [21, 22].

Our analysis also predicted ten novel isoforms for the *Peripherin 2* gene (*PRPH2),* half of them displaying > 5% contribution to overall gene expression. The PRPH2 protein is a cell-surface glycoprotein found in the outer segment of rod and cone photoreceptor cells for disk morphogenesis [23]. Defects in this gene can cause RP (OMIM: 608,133), macular dystrophy (OMIM: 169,150), adult vitelliform macular dystrophy (OMIM: 608,161), retinitis punctata albescens (OMIM: 136,880); central areolar choroidal dystrophy (OMIM: 613,105), and LCA (OMIM: 608,133) [24–26]. We validated isoform MSTRG.34897.3 (median TPM of 891.35; 12% contribution to expression levels) characterized by the skipping of exon 2 (Fig. 3c).

The KIAA1549 protein belongs to the UPF0606 family and is found at the connecting cilium of photoreceptor cells and synapses. Mutations in this gene are associated with autosomal recessive RP (OMIM: 618,613) [27]. Our data predicted the presence of 16 novel isoforms for the *KIAA1549* gene*,* ten of which are HEITs. We validated the isoform MSTRG.38096.12 (median TPM of 11.35 and a transcript contribution to overall gene expression of approximately 6%), which is characterized by the presence of a novel exon 12 (Fig. 3d).

The *Male Germ Cell Associated Kinase* (*MAK*) gene encodes a serine/threonine protein kinase essential for regulating the ciliary length of photoreceptors. Mutations in this gene are associated with autosomal recessive RP (OMIM: 614,181) [28]. We identified 21 novel isoforms for this gene, five of which are HEITs. We validated the transcript MSTRG.34222.19 (median TPM of 234.32 and a contribution to gene expression of 6.27%), characterized by an alternative 3’ coding exon (Fig. 3e).

The *Retinol Dehydrogenase 5* (*RDH5*) gene encodes an enzyme belonging to the short-chain dehydrogenases/reductases family. It catalyses the oxidation of the retinol isomers 11-cis-, 9-cis-, and 13-cis-retinol. Mutations in this gene cause a rare form of night blindness called fundus albipunctatus (OMIM: 136,880) [29, 30]. Read-through transcription has been previously reported between this gene and the upstream neighbouring gene *Biogenesis of Lysosomal Organelles Complex-1, Subunit 1* (*BLOC1S1*) [31]. We identified 14 novel isoforms in *RDH5,* three of which connect this gene with *BLOC1S1.* Among them, we validated the presence of transcript MSTRG.9138.5 (Fig. 3f), which has a median TPM of 15.74 and is predicted to represent 7.38%. of overall *RDH5* gene expression.

Finally, the *MER Proto-Oncogene Tyrosine Kinase* (*MERTK*) is a member of the MER/AXL/TYRO3 receptor kinase family that regulates rod outer segment fragment phagocytosis by the retinal pigment epithelium (RPE). Mutations in this gene cause autosomal recessive RP (OMIM: 613,862) [32, 33]. We identified 19 novel isoforms in *MERTK*, four of which contributed more than 5% to the overall gene expression. We validated the isoform MSTRG.23118.14, characterized by an alternative 5’ coding exon, an exon skipping event, and an intron retention (Fig. 3g). It has a median TPM value of 48.75 and a contribution of 10.77% to *MERTK* gene expression.

## Discussion

Over the past thirty years, basic research has generated remarkable progress in understanding the molecular basis of IRDs with a significant impact on translational applications to patients [34, 35]. Here, in an effort aimed at both paving the way towards the partial elucidation of the significant fraction of “missing heritability” still present in IRD patients and at gaining a more detailed understanding of the regulatory mechanisms underlying the expression of IRD genes, we performed a meta-analysis of bulk short-reads RNA-seq from the human retina by integrating the two largest, to our knowledge, freely available dataset collections [8, 9, 19]. This analysis led to the definition of the genomic organization of 218 genes responsible for monogenic forms of IRDs and to a better definition of alternative splicing events occurring in the human retina.

About 95% of multi-exon genes are alternatively spliced [36]. A systematic analysis of the extent of Alternative Splicing (AS), performed in a large number of tissues not including retina, revealed that most AS events are differentially regulated among tissues [37]. Tissues of neural origin, including the retina, have been reported to have more tissue-specific isoforms than non-neural tissues [38, 39]. However, transcriptome diversity among tissues and cell types is still poorly defined despite recent advances in sequencing technology.

We identified a large number of putative novel isoforms in the human retinal transcriptome. Our results suggest that the number of isoforms produced by IRD genes is higher than previously reported. We decided to focus our attention on the subset of transcripts likely to harbour the highest level of expression. Therefore, for each transcript we calculated the contribution to the overall expression of the corresponding IRD gene. To the best of our knowledge, this is the first time such analysis has been carried out systematically for IRD genes in the context of the human retina. These data are provided in a publicly accessible database along with the transcript expression ranking of all analysed IRD genes. As a result, we identified 936 transcripts, 435 of which are novel ones that display greater than 5% expression, termed as “highly expressed IRD transcripts” (HEITs). Among the novel HEITs, we found that novel exons and intron retentions were the most common features among these transcripts (223 and 90 events, respectively). In a few cases, we also found transcripts linking two genes. This phenomenon was previously reported as the generation of “chimaeras” (a term used for the fusion of two neighbouring genes that create a new transcript incorporating the sequences of both genes) and reported in around 65% of human RNAs by The Encyclopedia of DNA Elements (ENCODE) project [40]. These HEITs represent a reservoir of interesting transcripts that warrant further investigation to decipher their functional role in the retina.

We experimentally validated about 50% of selected HEITs by RT-PCR. However, we believe this validation rate is an underestimate due to the lower sensitivity of RT-PCR compared to RNA-seq. After all, the newly identified transcripts are supported by their presence across an extensive collection of RNA-seq datasets from a large number of individuals obtained in the context of two different experimental setups (i.e., TIGEM and NEI). Nevertheless, additional validation studies are necessary to further support the alternative IRD isoforms predicted by our analysis.

Our reported meta-analysis gives an overview of novel isoforms present in physiological conditions of the human retina, highlighting the diversity and specificity of retinal transcripts. One limitation of our study is the short length of RNA-seq reads (< 300 bp). Long-read sequencing is emerging as an alternative, powerful approach to identify full-length isoforms and simultaneously define their transcription start site, splice sites, and the polyA site. This technology has been successfully used in the human retina to describe a novel isoform of the *CRB1* gene [41]. Further studies of the human retina transcriptome based on long-read sequencing are required to expand the findings described herein. Moreover, single-cell RNA sequencing (scRNA-seq)-based strategies can further increase the resolution of AS events to single retinal cell types. Such approaches have already been applied to the human retina in physiological and pathological conditions [42–45]. Combining the above-mentioned approaches can further delineate the complexity of the human retinal transcriptome.

## Conclusions

To the best of our knowledge, this is the most comprehensive and extended meta-analysis of IRD genes carried out on RNA-Seq data in the human retina. Our work yielded a reliable quantification of IRD transcript expression in this tissue, including the identification of novel ones. The generated resource can improve our understanding of the organization of the transcriptional units of IRD genes and, ultimately, of the molecular mechanisms underlying inherited retinal diseases. The identification of putative novel isoforms can address at least a fraction of the cases of “missing heritability” that are observed in diagnostic processes of IRDs based on genomic NGS approaches. Combined with Whole Genome Sequencing that extends beyond the protein-coding regions, it can help uncover and interpret variants in regulatory regions.

## Methods

### RNA-seq data

A total of 177 bulk RNA-seq datasets from human retinas of non-visually impaired retinal post-mortem donors were retrieved from two different sources: the first one (hereafter labelled as TIGEM) contains data from 50 human retina samples [8], and the second dataset (hereafter labelled as NEI) contains 127 samples [9, 19].

### Data analysis

For our analysis, we selected samples with a reported RNA integrity number (RIN) > 5.0 to assure the use of high-quality data. Raw RNA-seq reads were trimmed for Illumina adapters and low quality in TrimGalore! (v0.41) [46]. Quality control check was performed using FastQC (v0.11.5) [47]. Trimmed reads were aligned to the GRCh38v.98 human genome using the 2-pass mapping strategy from STAR (v2.7.2a) [48]. Samples with less than 10 million mapped reads and/or less than 70% of reads aligned to the reference genome were removed.

### Transcript identification

RNA-seq alignments were assembled into potential transcripts at a single sample level with the Reference Annotation-based Transcript Assembly method using StringTie (v2.1.1) [49]. A total of 161 sample-level predictions from the NEI and TIGEM datasets were merged using the "*merge*" option from StringTie to create a single set of assembled transcripts and identify putative novel ones with a minimum length of 50 nucleotides.

FASTA files were created using the transcript-assembled file from StringTie. Transcript quantification was calculated per sample using Salmon (v1.6.0) [50], adding *–seqBias –dumpEq –useVBOpt* options to the default parameters. Isoforms with zero counts across samples were discarded. Transcripts lengths and abundance estimates were imported to R using the tximport package (v1.16.1) [51]. Transcripts with a value higher than one median Transcript per Million (TPM) were retained for further analysis.

To assess the putative biological relevance of all the IRD transcripts, we estimated the contribution of each transcript to the overall gene expression. Transcripts contributing more than 5% to the overall expression of the corresponding gene were kept for further analysis.

### Coding potential of novel isoforms

We predicted Open Reading Frames (ORF) embedded within the novel transcripts using EMBOSS:*getorf* (v.6.6.0.0) [52] with a minimum sequence length of 200 nucleotides and "ATG" as initiation codon. All the sequences predicted to be encoded by the identified ORFs were analysed by BLAT (BLAST-like alignment tool) [53] to determine if any sequence led to the expansion of the already annotated ORF. Furthermore, the predicted sequences were also compared against the non-redundant protein (nr) database by Protein Basic Local Alignment Search Tool (BLASTP) [54, 55].

Protein databases, like HMMER [56] and the Conserved Domains Database (CDD) [57] were used to study the protein changes produced by the novel proteins that were predicted to alter the known ORF. For a list of 12 novel transcripts, protein models were generated using AlphaFold2 and ColabFold [58, 59]. The selected sub-model was ranked as the best for each gene according to the program prediction and was visualized using PyMol (v2.5.2) [60].

### Reverse Transcriptase (RT-) PCR validation

We selected a subset of newly-identified candidate transcripts of IRD genes for independent validation by RT-PCR. Transcript-specific primers were designed using the freely available tools OligoCalc [61] and Primer3Plus [62]. RT-PCR was performed using the AmpliTaq Gold DNA Polymerase kit (Applied Biosystems) on human retinas, podocytes, fibroblasts, and blood samples. Retina samples were obtained from non-visually impaired post-mortem donors [63]. Total RNA extraction was performed using the miRNeasy kit (QIAGEN), and cDNA production was obtained using the QuantiTect Reverse Transcription Kit (QIAGEN), following the manufacturer's protocol. Blood samples were collected and stored in Tempus™ Blood RNA Tube (Applied Biosystems TM), and RNA was extracted using Tempus Spin RNA Isolation Kit (Applied Biosystems TM). RT-PCR products underwent Sanger sequencing to confirm their identity. Oligonucleotides sequences are reported in Supplementary Table S1.

## Supplementary Information

**Additional file 1: Supplementary Dataset File 1.** List of the IRD genes analyzed in this study.

**Additional file 2: Supplementary Dataset File 2.** List of all IRD transcripts with more than 1 median TPM.

**Additional file 3: Supplementary Dataset File 3.** Summary of transcripts identified for each IRD gene.

**Additional file 4: Supplementary Dataset File 4.** Overall contribution to gene expression of annotated and novel transcripts with more than 1 median TPM for each IRD gene.

**Additional file 5: Supplementary Dataset File 5.** Annotated transcripts with a) more than 1 median TPM and b) relative contribution to gene expression greater than 5%.

**Additional file 6:Supplementary Dataset File 6.** Putative novel IRD transcripts with a) more than 1 median TPM and b) relative contribution to overall expression of the corresponding gene greater than 5%.

**Additional file 7: Supplementary Table S1.** Oligonucleotides primers used in this study. **Figure S1.** Predicted 3D protein structures encoded by the novel HEITs. **Figure S2.** Source data for Fig. 3.

## Data Availability

The RNA-seq datasets analysed during the current study and supporting the conclusions of this article are available in the ENA repository at: PRJEB42859 and in the GEO: GSE115828 and are included in the following published articles: Pinelli M, Carissimo A, Cutillo L, Lai CH, Mutarelli M, Moretti MN, et al. An atlas of gene expression and gene co-regulation in the human retina. Nucleic Acids Res. 2016;44:5773–84, https://doi.org/10.1093/nar/gkw486. Ratnapriya R, Sosina OA, Starostik MR, Kwicklis M, Kapphahn RJ, Fritsche LG, et al. Retinal transcriptome and eQTL analyses identify genes associated with age-related macular degeneration. Nat Genet. 2019;51:606–10, https://doi.org/10.1038/s41588-019-0351-9 Brooks MJ, Chen HY, Kelley RA, Mondal AK, Nagashima K, De Val N, et al. Improved Retinal Organoid Differentiation by Modulating Signaling Pathways Revealed by Comparative Transcriptome Analyses with Development In Vivo. Stem Cell Reports. 2019;13:891–905, https://doi.org/10.1016/j.stemcr.2019.09.009 Data reported in this paper are publicly available [https://retina.tigem.it/retina_disease_gene.php]. This database provides all the results, including TPM values, the relative contribution to the overall gene expression levels, and the genomic coordinates of the transcripts. All analyses conducted are outlined in the Methods section, including software version(s) used. Example code is available upon request.

## Abbreviations

IRD: Inherited retinal diseases
NGS: Next-Generation Sequencing
RNA-seq: RNA-sequencing
AS: Alternative splicing
USH: Usher syndrome
RP: Retinitis Pigmentosa
LCA: Leber Congenital Amaurosis
HEITs: Highly Expressed IRD Transcripts
ORF: Open Reading Frame
ENCODE: The Encyclopedia of DNA Elements
scRNA-seq: Single-cell RNA sequencing
TPM: Transcript per Million
