# Supplementary material for: Definition of the transcriptional units of inherited retinal disease genes by meta-analysis of human retinal transcriptome data
Source: BMC Genomics. 2023 Apr 18;24:206. doi: 10.1186/s12864-023-09300-w (PMC10111803; doi:10.1186/s12864-023-09300-w)
Supplement: Supplementary file 7 — Additional file 7: Supplementary Table S1. Oligonucleotides primers used in this study. Figure S1. Predicted 3D protein structures encoded by the novel HEITs. Figure S2. Source data for Fig. 3. [file 12864_2023_9300_MOESM7_ESM.pdf]

Supplementary Table S1

Oligonucleotides primers used in this study:

Oligonucleotide name	Oligonucleotide Forward sequence	Oligonucleotide Reverse sequence	Annealing temperature
PRPH2	AACAACGGTTTTCGGGACTGG	CGCAGCCCAATTGTAATGGTC	56 °C
GRK1	AGCTGTATGCCTGCAAGAAGC	TGGAGTTCAGTTTGGTGACGG	56 °C
MERTK	GAATGAAATAGGCTGGTCTGC	GGAGTCAACAGTAGAAGAGAG	56 °C
hybrid RDHBLO	GGAGAACTTCAACCAGGCACT	TTCTCCAGACTCTCCAGGTTG	56 °C
KIAA1549	TCCCTCAGATGCTGACTCTAC	GTGGGATCACTTTCCTCCAG	56 °C
MAK	CAGAGGATCCACTTAGCACCT	CCATGATTGCATCCCTGCACT	56 °C
PLA2G5	TGCCCAAGGAAGTTGCTCATG	TTGTGTTGCAATGTCTCTCCC	56 °C

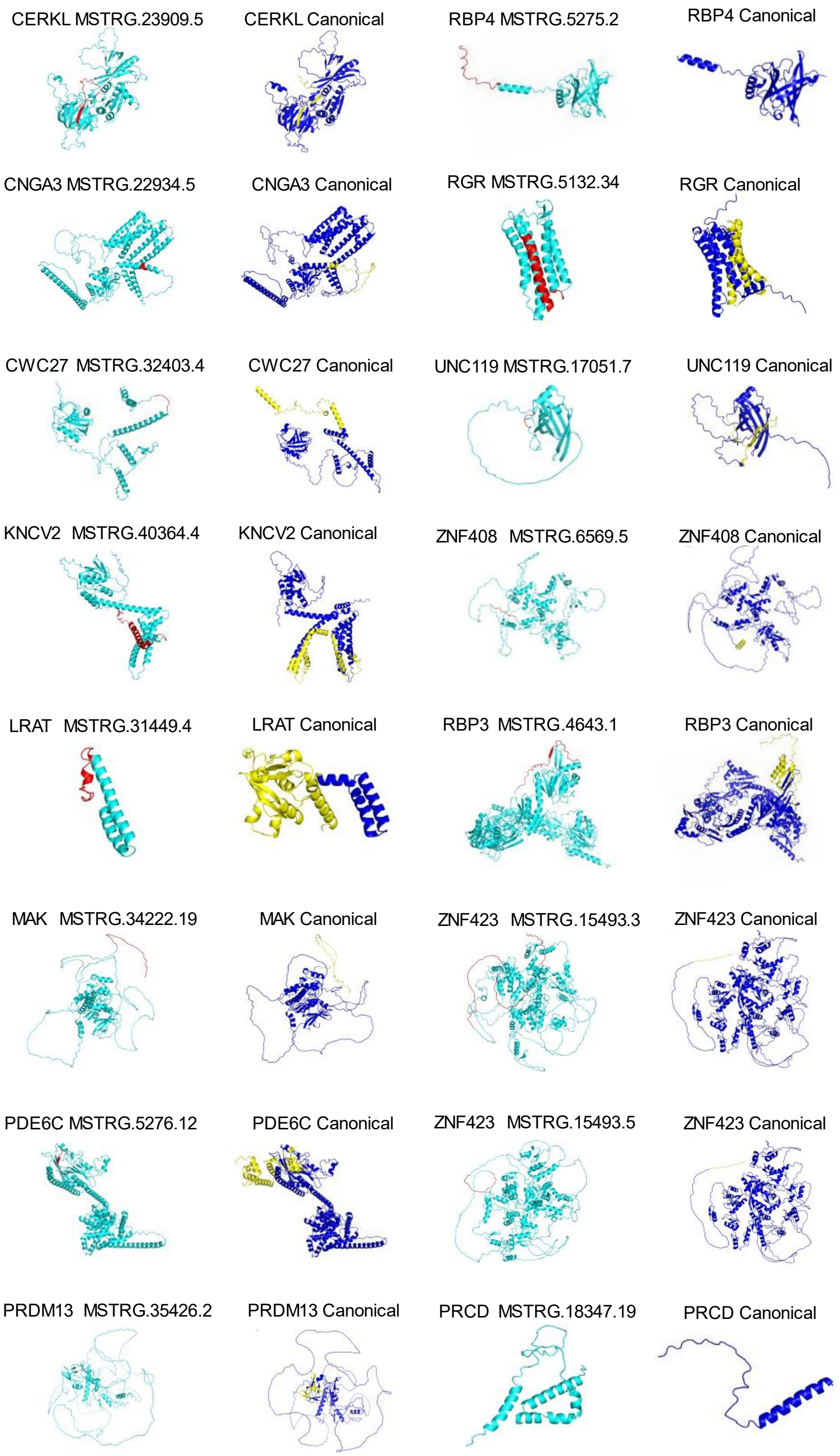

Figure S1. Predicted 3D protein structures encoded by the novel HEITs (light blue) compared with their corresponding canonical forms (dark blue). Protein modelling was carried out with AlphaFold2 and ColabFold. The distinguishing features between the novel and the canonical proteins are highlighted in red and yellow, respectively.

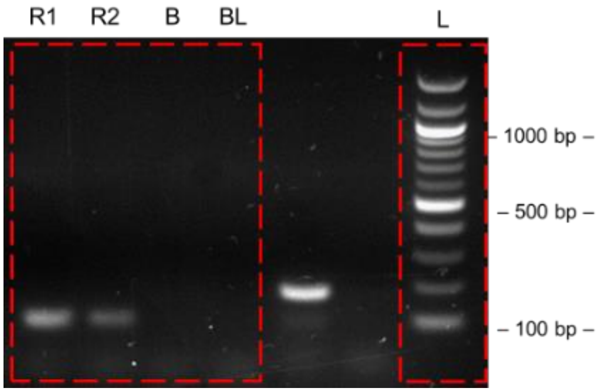

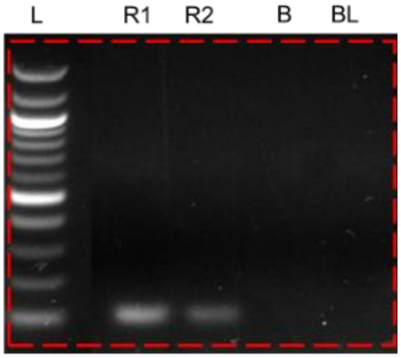

Source data

Fig 3b - *GRK1*

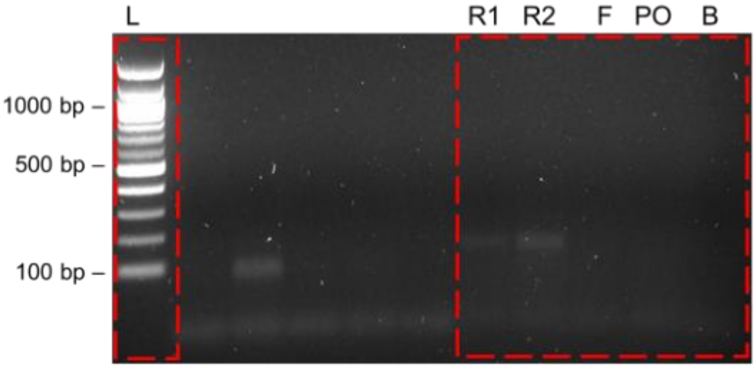

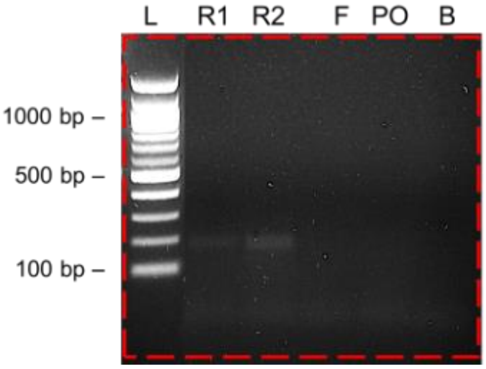

Source data

Fig 3d - *KIAA1549*

Figure S2. Source data for Figure 3.
